# One-pot synthesis of (1*RS*,21*SR*)-diethyl 2-[23-amino-22-eth­oxy­carbonyl-8,11,14-trioxa-25-aza­tetra­cyclo­[19.3.1.0^2,7^.0^15,20^]penta­cosa-2,4,6,15(20),16,18,22-heptaen-25-yl]but-2-endioate

**DOI:** 10.1107/S205698901801160X

**Published:** 2018-08-21

**Authors:** Thi Thanh Van Tran, Tuan Anh Le, Hong Hieu Truong, Thi Nhung Dao, Anatoly T. Soldatenkov, Victor N Khrustalev

**Affiliations:** a Faculty of Chemistry, University of Science, Vietnam National University, 19 Le Thanh Tong, Hoan Kiem, Hanoi, Vietnam; b Organic Chemistry Department, Peoples’ Friendship University of Russia (RUDN University), 6 Miklukho-Maklay St., Moscow 117198, Russian Federation; c Inorganic Chemistry Department, Peoples’ Friendship University of Russia (RUDN University), 6 Miklukho-Maklay St., Moscow 117198, Russian Federation; d National Research Centre ‘Kurchatov Institute’, 1 Acad. Kurchatov Sq., Moscow 123182, Russian Federation

**Keywords:** macroheterocycles, aza­crown ether, one-component reaction, cytotoxicity, anti­cancer activity, crystal structure

## Abstract

The title compound, C_30_H_34_N_2_O_9_ (**4**), is a product of the Michael reaction of aza­crown ether with dimethyl acetyl­enedi­carboxyl­ate modified by an addition of NH_3_ (aq.) at 298 K. The aza-14-crown-4-ether ring adopts a bowl conformation. The dihedral angle between the planes of the benzene rings fused to the aza-14-crown-4-ether moiety is 8.65 (5)°. The tetra­hydro­pyridine ring has a boat conformation. The mol­ecular conformation is supported by one N—H⋯O and two C—H⋯O intra­molecular hydrogen bonds. Both heterocyclic and amino N atoms have essentially planar configurations (sums of the bond angles are 359.35 and 358.00°). Compound **4** crystallizes as a racemate consisting of enanti­omeric pairs of the 1*R*,21*S* diastereomer. In the crystal, mol­ecules of **4** are connected by N—H⋯O hydrogen bonds, forming chains along [100]. According to the *PASS* program (computer prediction of biological activities), compound **4** may exhibit anti­allergic (72% probability) and anti­asthmatic (67%) activity, as well as be a membrane permeability inhibitor (65%).

## Abstract 2

A new macrocycle obtained by the Michael reaction of aza­crown ether with dimethyl acetyl­enedi­carboxyl­ate was studied by X-ray diffraction, and its potential bioactivity was estimated from the structural data.

## Chemical context   

Over the last several decades, aza­crown ethers have been designed, synthesized and applied as macrocyclic ligands for coordination chemistry (Hiraoka, 1978 ▸; Pedersen, 1988 ▸; Schwan & Warkentin, 1988 ▸; Gokel & Murillo, 1996 ▸; Bradshaw & Izatt, 1997 ▸). Recently, we have developed effective new methods for the synthesis of aza­crown ethers containing the heterocyclic subunits piperidine (Levov *et al.*, 2006 ▸, 2008*a* ▸, *b* ▸; Anh *et al.*, 2008 ▸, 2012*a* ▸,*b* ▸,*c* ▸; Hieu *et al.* 2012*a* ▸,*b* ▸, 2013*a* ▸), per­hydro­pyrimidine (Hieu *et al.*, 2011 ▸), perhydro­triazine (Khieu *et al.*, 2011 ▸), pyridine (Le *et al.*, 2014 ▸; Tuan *et al.*, 2015 ▸; Anh *et al.*, 2018 ▸) and bis­pyridine (Komarova *et al.*, 2008 ▸; Sokol *et al.*, 2011 ▸). These new aza­crown compounds also are inter­esting as potential anti­cancer agents because of their cytotoxicity (Le *et al.*, 2014 ▸; Le *et al.*, 2015 ▸; Ahn *et al.*, 2018 ▸).

**Figure 1:**
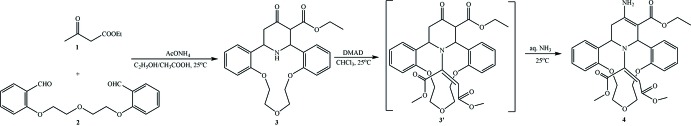
The modified reaction yielding the γ-amino-*N*-propyl­piperidine **4**.

**Figure 2:**
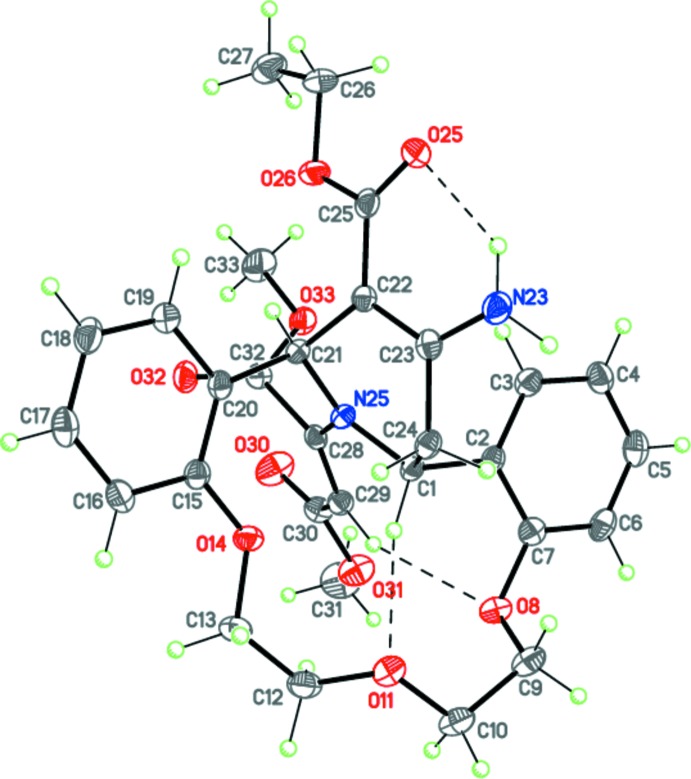
The mol­ecular structure of **4**. Displacement ellipsoids are shown at the 50% probability level. H atoms are presented as small spheres of arbitrary radius. Dashed lines indicate the intra­molecular N—H⋯O and C—H⋯O hydrogen bonds.

In our previous work, we have studied the Michael addition of aza­crown ethers to dimethyl acetyl­enedi­carboxyl­ate (Anh *et al.*, 2012*a* ▸,*b* ▸; Hieu *et al.* 2013*a* ▸,*b* ▸). We have also found recently that the expected *N*-vynilation proceeded smoothly with the formation of an *N-*maleinate derivative of the aza­crown system. Modification of the reaction by the addition of NH_3_ (aq.) and continuous stirring for three days at 298 K produced the unexpected γ-amino-*N*-propyl­piperidine (**4**) in a yield of 40% (Fig. 1 ▸). According to the PASS program (Prediction of Activity Spectra for Substances – *i.e.* computer prediction of biological activities**;** Sadym *et al.*, 2003 ▸), the title compound has the potential to exhibit anti­allergic (72% probability), anti­asthmatic (67%) and membrane permeability inhibiting (65%) activities. The obtained compound was studied by X-ray diffraction analysis (Fig. 2 ▸).
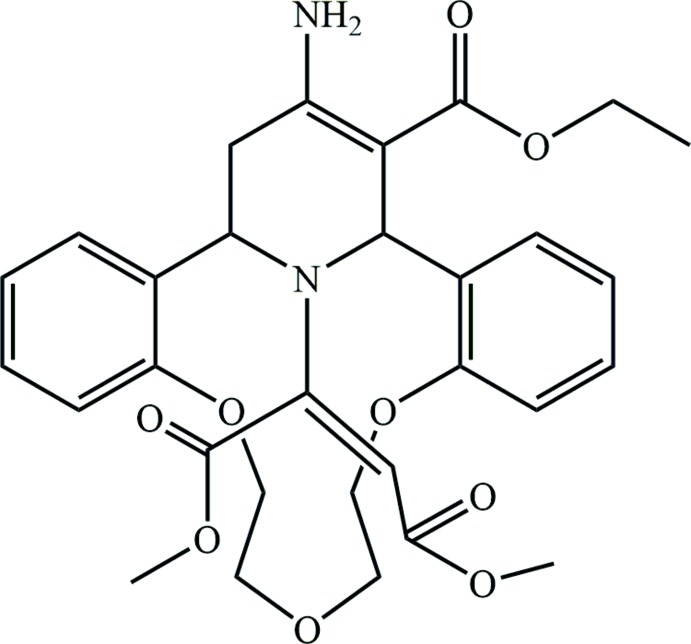
.

## Structural commentary   

The mol­ecule of **4**, C_30_H_34_N_2_O_9_, comprises a fused tetra­cyclic system containing the aza-14-crown-3-ether macrocycle, one piperidine and two benzene rings (Fig. 2 ▸). The aza-14-crown-3-ether ring adopts a bowl conformation. The configuration of the C7—O8—C9—C10 —O11—C12—C13—O14—C15 polyether chain is *t*—*g*(−)—*g*(−)—*t*—*g*(+)—*t* (*t* = *trans*, 180°; *g* = *gauche*, ±60°). The dihedral angle between the planes of the benzene rings fused to the aza-14-crown-4-ether moiety is 8.65 (5)°. The tetra­hydro­pyridine ring adopts a boat conformation. The conformations of the aza-14-crown-4-ether and piperidine rings are supported by the three intra­molecular (one N—H⋯O and two C—H⋯O) hydrogen bonds (Table 1 ▸). The nitro­gen N23 and N25 atoms have practically planar geometries (the sums of the bond angles are 359.35 and 358.00°, respectively).

The mol­ecule of **4** possesses two asymmetric centers at the C1 and C21 carbon atoms and potentially can have four diastereomers. The crystal of **4** is racemic and consists of enanti­omeric pairs with the following relative configuration of the centers: 1*R*,21*S*.

## Supra­molecular features   

**Figure 3:**
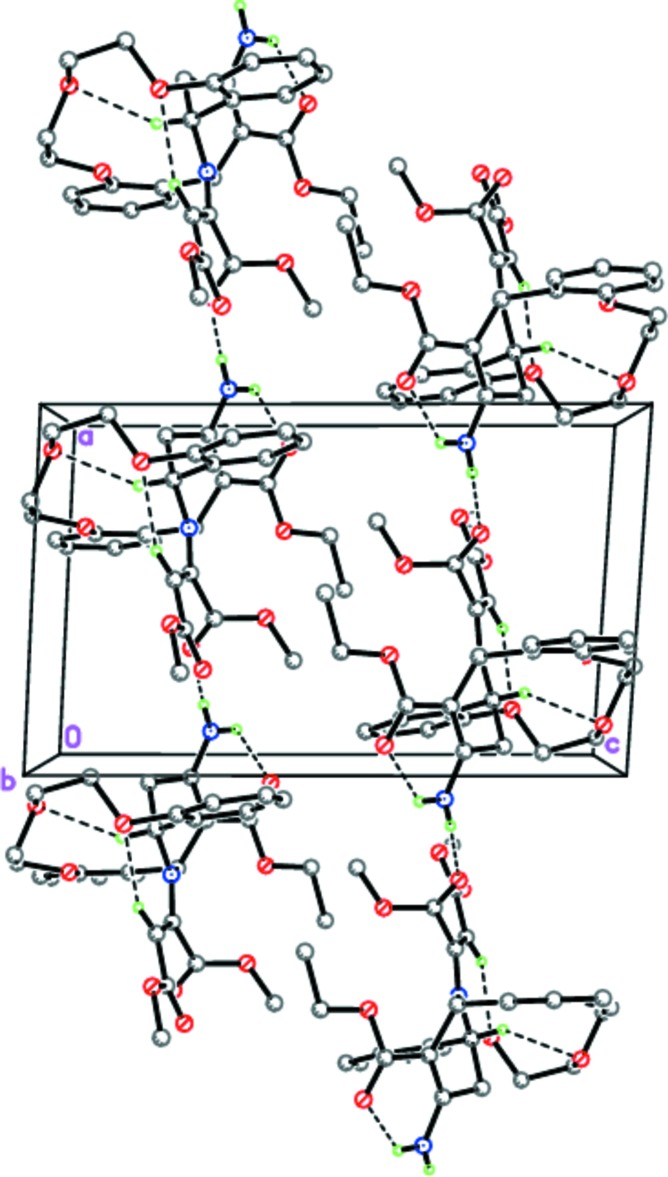
The hydrogen-bonded chains of **4** along the *a* axis. Dashed lines indicate the intra­molecular N—H⋯O and C—H⋯O and the inter­molecular N—H⋯O hydrogen bonds.

In the crystal, mol­ecules of **4** form hydrogen-bonded chains propagating along [100] through strong inter­molecular N—H⋯O hydrogen bonds (Fig. 3 ▸, Table 1 ▸). The chains are stacking along the *b-*axis direction.

## Synthesis and crystallization   

A solution of 1.30 g (10.00 mmol) of ethyl aceto­acetate (**1**), 3.14 g (10.00 mmol) of 1,5-bis-(2-formyl­phen­oxy)-3-oxaopetane (**2**) and 1.00 g (13.00 mmol) of ammonium acetate in a mixture of 30 ml ethanol and 1 ml acetic acid was stirred at 298 K. The reaction was monitored by TLC and found to be complete after 6 h. The reaction mixture was allowed to cool to room temperature before being neutralized with sodium carbonate solution; the product was then extracted with chloro­form (3 × 50 ml). By TCL, compound **3** was determined to be successfully synthesized. The solvent (CDCl_3_) was evaporated under vacuum until 30ml of CDCl_3_ was left, 1.42 g (10 mmol) of DMAD was added and the solution was stirred for 30 minutes at 298 K. Then NH_3_ (aq.) was added to the reaction mixture, which was stirred continuously. After three days, the residue was purified by column chromatography and recrystallized from ethanol to obtain 2.27 g of the pure aza­crown ether **4** as light-yellow crystals (yield 60%). *T*_m_ = 525–526 K. *R*_f_ = 0.85 [*n*-hexa­ne:ethyl acetate = 1:1 (*v*:*v*)].

## Refinement   

Crystal data, data collection and structure refinement details are summarized in Table 2 ▸. The hydrogen atoms of the amino group were localized in difference-Fourier maps and refined isotropically with constrained thermal displacement parameters [*U*_iso_(H = 1.2*U*_eq_(N)]. Other hydrogen atoms were placed in calculated positions with C—H bond lengths of 0.95–1.00 Å and refined using a riding model with constrained isotropic displacement parameters [*U*_iso_(H) = 1.5*U*_eq_(C) for the CH_3_ groups and 1.2*U*_eq_(C) for all others].

## Supplementary Material

Crystal structure: contains datablock(s) I. DOI: 10.1107/S205698901801160X/ld2144sup1.cif

Structure factors: contains datablock(s) I. DOI: 10.1107/S205698901801160X/ld2144Isup2.hkl

Supporting information file. DOI: 10.1107/S205698901801160X/ld2144Isup3.cml

CCDC reference: 1862265

Additional supporting information:  crystallographic information; 3D view; checkCIF report

## Figures and Tables

**Table 1:** Hydrogen-bond geometry (Å, °)

*D*—H⋯*A*	*D*—H	H⋯*A*	*D*⋯*A*	*D*—H⋯*A*
C1—H1⋯O11	1.00	2.38	3.2572 (17)	146
N23—H23*A*⋯O25	0.891 (18)	2.040 (18)	2.7127 (17)	131.4 (15)
N23—H23*B*⋯O32^i^	0.936 (18)	2.063 (18)	2.9986 (17)	176.7 (16)
C29—H29⋯O8	0.95	2.44	3.3439 (17)	159

Symmetry code: (i) 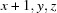.

**Table 2:** Experimental details

Crystal data	
Chemical formula	C_30_H_34_N_2_O_9_
*M*_r_	566.59
Crystal system, space group	Triclinic, *P* 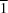
Temperature (K)	120
*a*, *b*, *c* (Å)	9.1172 (9), 10.3752 (10), 14.7482 (14)
α, β, γ (°)	89.044 (2), 86.658 (2), 82.896 (2)
*V* (Å^3^)	1382.0 (2)
*Z*	2
Radiation type	Mo *K*α
μ (mm^−1^)	0.10
Crystal size (mm)	0.25 × 0.25 × 0.05

Data collection	
Diffractometer	Bruker APEXII CCD
Absorption correction	Multi-scan (*SADABS*; Sheldrick, 2003 ▸)
*T*_min_, *T*_max_	0.969, 0.990
No. of measured, independent and observed [*I* > 2σ(*I*)] reflections	27765, 10063, 6502
*R*_int_	0.046
(sin θ/λ)_max_ (Å^−1^)	0.761

Refinement	
*R*[*F* ^2^ > 2σ(*F* ^2^)], *wR*(*F* ^2^), *S*	0.055, 0.127, 1.03
No. of reflections	10063
No. of parameters	379
H-atom treatment	H atoms treated by a mixture of independent and constrained refinement
Δρ_max_, Δρ_min_ (e Å^−3^)	0.39, −0.25

Computer programs: *APEX2* (Bruker, 2005 ▸), *SAINT* (Bruker, 2001 ▸) and *SHELXTL* (Sheldrick, 2015 ▸).

## supplementary crystallographic information

### Crystal data

**Table:**

C_30_H_34_N_2_O_9_	*Z* = 2
*M**_r_* = 566.59	*F*(000) = 600
Triclinic, *P*1	*D*_x_ = 1.362 Mg m^−^^3^
*a* = 9.1172 (9) Å	Mo *K*α radiation, λ = 0.71073 Å
*b* = 10.3752 (10) Å	Cell parameters from 5654 reflections
*c* = 14.7482 (14) Å	θ = 2.4–32.2°
α = 89.044 (2)°	µ = 0.10 mm^−^^1^
β = 86.658 (2)°	*T* = 120 K
γ = 82.896 (2)°	Plate, yellow
*V* = 1382.0 (2) Å^3^	0.25 × 0.25 × 0.05 mm

### Data collection

**Table:**

Bruker APEXII CCD diffractometer	6502 reflections with *I* > 2σ(*I*)
Radiation source: fine-focus sealed tube	*R*_int_ = 0.046
φ and ω scans	θ_max_ = 32.8°, θ_min_ = 2.0°
Absorption correction: multi-scan (*SADABS*; Sheldrick, 2003)	*h* = −13→13
*T*_min_ = 0.969, *T*_max_ = 0.990	*k* = −15→15
27765 measured reflections	*l* = −22→22
10063 independent reflections	

### Refinement

**Table:**

Refinement on *F*^2^	Primary atom site location: difference Fourier map
Least-squares matrix: full	Secondary atom site location: difference Fourier map
*R*[*F*^2^ > 2σ(*F*^2^)] = 0.055	Hydrogen site location: mixed
*wR*(*F*^2^) = 0.127	H atoms treated by a mixture of independent and constrained refinement
*S* = 1.03	*w* = 1/[σ^2^(*F*_o_^2^) + (0.0509*P*)^2^ + 0.1608*P*] where *P* = (*F*_o_^2^ + 2*F*_c_^2^)/3
10063 reflections	(Δ/σ)_max_ < 0.001
379 parameters	Δρ_max_ = 0.39 e Å^−^^3^
0 restraints	Δρ_min_ = −0.25 e Å^−^^3^

### Special details

**Table:**

Geometry. All esds (except the esd in the dihedral angle between two l.s. planes) are estimated using the full covariance matrix. The cell esds are taken into account individually in the estimation of esds in distances, angles and torsion angles; correlations between esds in cell parameters are only used when they are defined by crystal symmetry. An approximate (isotropic) treatment of cell esds is used for estimating esds involving l.s. planes.

### Fractional atomic coordinates and isotropic or equivalent isotropic displacement parameters (Å^2^)

**Table:**

	*x*	*y*	*z*	*U*_iso_*/*U*_eq_	
C1	0.81612 (14)	0.57839 (12)	0.20889 (9)	0.0141 (2)	
H1	0.7967	0.6281	0.1513	0.017*	
C2	0.85575 (14)	0.67267 (13)	0.27875 (9)	0.0155 (3)	
C3	0.87367 (16)	0.63719 (14)	0.36921 (9)	0.0197 (3)	
H3	0.8576	0.5523	0.3891	0.024*	
C4	0.91474 (16)	0.72407 (15)	0.43085 (10)	0.0227 (3)	
H4	0.9285	0.6977	0.4921	0.027*	
C5	0.93576 (17)	0.84913 (15)	0.40326 (11)	0.0242 (3)	
H5	0.9630	0.9087	0.4456	0.029*	
C6	0.91694 (16)	0.88691 (14)	0.31367 (11)	0.0224 (3)	
H6	0.9302	0.9728	0.2945	0.027*	
C7	0.87861 (15)	0.79878 (13)	0.25201 (10)	0.0179 (3)	
O8	0.85439 (11)	0.83852 (10)	0.16315 (7)	0.0210 (2)	
C9	0.98394 (17)	0.82770 (15)	0.10229 (10)	0.0247 (3)	
H9A	1.0559	0.7541	0.1210	0.030*	
H9B	1.0318	0.9082	0.1035	0.030*	
C10	0.93712 (18)	0.80584 (14)	0.00820 (10)	0.0250 (3)	
H10A	0.8532	0.8716	−0.0062	0.030*	
H10B	1.0202	0.8151	−0.0367	0.030*	
O11	0.89354 (11)	0.67802 (10)	0.00304 (7)	0.0223 (2)	
C12	0.75156 (17)	0.67584 (15)	−0.03119 (10)	0.0239 (3)	
H12A	0.7496	0.7151	−0.0928	0.029*	
H12B	0.6758	0.7276	0.0085	0.029*	
C13	0.71654 (17)	0.53812 (15)	−0.03494 (9)	0.0224 (3)	
H13A	0.6301	0.5342	−0.0720	0.027*	
H13B	0.8020	0.4821	−0.0635	0.027*	
O14	0.68447 (11)	0.49285 (9)	0.05566 (6)	0.0192 (2)	
C15	0.66801 (15)	0.36325 (13)	0.06612 (9)	0.0169 (3)	
C16	0.65128 (16)	0.28520 (14)	−0.00827 (10)	0.0204 (3)	
H16	0.6523	0.3217	−0.0678	0.024*	
C17	0.63327 (17)	0.15515 (15)	0.00416 (10)	0.0239 (3)	
H17	0.6228	0.1030	−0.0468	0.029*	
C18	0.63047 (17)	0.10129 (15)	0.09031 (11)	0.0243 (3)	
H18	0.6174	0.0124	0.0992	0.029*	
C19	0.64699 (16)	0.17908 (14)	0.16373 (10)	0.0207 (3)	
H19	0.6451	0.1416	0.2229	0.025*	
C20	0.66621 (14)	0.31004 (13)	0.15426 (9)	0.0160 (3)	
C21	0.68462 (15)	0.37961 (13)	0.24427 (9)	0.0147 (2)	
H21	0.5991	0.3621	0.2863	0.018*	
C22	0.82317 (14)	0.32219 (13)	0.28954 (9)	0.0151 (3)	
C23	0.95330 (15)	0.36568 (13)	0.25977 (9)	0.0161 (3)	
N23	1.08748 (14)	0.32444 (13)	0.29035 (9)	0.0211 (3)	
H23A	1.0903 (19)	0.2647 (17)	0.3346 (12)	0.025*	
H23B	1.1662 (19)	0.3727 (17)	0.2762 (12)	0.025*	
C24	0.94479 (15)	0.47044 (13)	0.18843 (9)	0.0163 (3)	
H24A	0.9322	0.4317	0.1289	0.020*	
H24B	1.0391	0.5089	0.1841	0.020*	
N25	0.68040 (12)	0.52165 (11)	0.23838 (7)	0.0142 (2)	
C25	0.81729 (16)	0.23578 (13)	0.36705 (9)	0.0173 (3)	
O25	0.92621 (11)	0.17777 (10)	0.40161 (7)	0.0217 (2)	
O26	0.67790 (11)	0.22664 (10)	0.40149 (7)	0.0211 (2)	
C26	0.66542 (18)	0.14964 (15)	0.48444 (10)	0.0249 (3)	
H26A	0.7269	0.1797	0.5311	0.030*	
H26B	0.6991	0.0568	0.4722	0.030*	
C27	0.50501 (18)	0.16773 (16)	0.51650 (11)	0.0285 (3)	
H27A	0.4906	0.1141	0.5708	0.043*	
H27B	0.4451	0.1416	0.4685	0.043*	
H27C	0.4745	0.2593	0.5311	0.043*	
C28	0.55098 (14)	0.60130 (13)	0.24700 (9)	0.0149 (2)	
C29	0.53278 (15)	0.72930 (13)	0.22462 (9)	0.0171 (3)	
H29	0.6112	0.7662	0.1926	0.020*	
C30	0.39565 (16)	0.81067 (14)	0.24871 (10)	0.0206 (3)	
O30	0.28235 (12)	0.77676 (11)	0.28223 (9)	0.0332 (3)	
O31	0.41022 (12)	0.93716 (10)	0.22944 (8)	0.0281 (3)	
C31	0.2821 (2)	1.02831 (17)	0.25180 (14)	0.0377 (4)	
H31A	0.3066	1.1169	0.2416	0.057*	
H31B	0.2501	1.0171	0.3157	0.057*	
H31C	0.2020	1.0131	0.2133	0.057*	
C32	0.42192 (15)	0.53990 (13)	0.29146 (9)	0.0167 (3)	
O32	0.33589 (11)	0.48529 (10)	0.25174 (7)	0.0235 (2)	
O33	0.42394 (11)	0.55003 (10)	0.38109 (6)	0.0208 (2)	
C33	0.30089 (19)	0.50518 (18)	0.43345 (11)	0.0324 (4)	
H33A	0.3098	0.5218	0.4980	0.049*	
H33B	0.3016	0.4118	0.4243	0.049*	
H33C	0.2079	0.5516	0.4135	0.049*	

### Atomic displacement parameters (Å^2^)

**Table:**

	*U*^11^	*U*^22^	*U*^33^	*U*^12^	*U*^13^	*U*^23^
C1	0.0126 (6)	0.0147 (6)	0.0152 (6)	−0.0042 (5)	0.0011 (5)	0.0000 (5)
C2	0.0119 (6)	0.0172 (6)	0.0177 (6)	−0.0033 (5)	0.0017 (5)	−0.0022 (5)
C3	0.0198 (7)	0.0204 (7)	0.0192 (7)	−0.0050 (5)	0.0008 (5)	−0.0006 (5)
C4	0.0217 (7)	0.0276 (8)	0.0193 (7)	−0.0042 (6)	−0.0022 (5)	−0.0039 (6)
C5	0.0211 (7)	0.0251 (8)	0.0274 (8)	−0.0048 (6)	−0.0023 (6)	−0.0085 (6)
C6	0.0208 (7)	0.0160 (7)	0.0307 (8)	−0.0040 (5)	0.0003 (6)	−0.0040 (6)
C7	0.0153 (6)	0.0180 (7)	0.0200 (7)	−0.0007 (5)	0.0007 (5)	−0.0008 (5)
O8	0.0222 (5)	0.0191 (5)	0.0211 (5)	−0.0021 (4)	0.0026 (4)	0.0027 (4)
C9	0.0249 (7)	0.0239 (8)	0.0257 (8)	−0.0082 (6)	0.0058 (6)	0.0018 (6)
C10	0.0305 (8)	0.0208 (7)	0.0239 (7)	−0.0077 (6)	0.0047 (6)	0.0028 (6)
O11	0.0234 (5)	0.0195 (5)	0.0241 (5)	−0.0038 (4)	0.0012 (4)	0.0010 (4)
C12	0.0246 (7)	0.0262 (8)	0.0207 (7)	−0.0029 (6)	−0.0009 (6)	0.0068 (6)
C13	0.0256 (7)	0.0277 (8)	0.0143 (6)	−0.0064 (6)	0.0000 (5)	0.0044 (5)
O14	0.0243 (5)	0.0198 (5)	0.0139 (5)	−0.0053 (4)	0.0002 (4)	0.0014 (4)
C15	0.0141 (6)	0.0191 (7)	0.0178 (6)	−0.0031 (5)	−0.0005 (5)	−0.0012 (5)
C16	0.0185 (7)	0.0266 (7)	0.0166 (6)	−0.0038 (6)	−0.0021 (5)	−0.0028 (5)
C17	0.0233 (7)	0.0268 (8)	0.0228 (7)	−0.0055 (6)	−0.0031 (6)	−0.0091 (6)
C18	0.0266 (8)	0.0183 (7)	0.0292 (8)	−0.0058 (6)	−0.0052 (6)	−0.0043 (6)
C19	0.0222 (7)	0.0199 (7)	0.0209 (7)	−0.0054 (5)	−0.0049 (5)	0.0010 (5)
C20	0.0135 (6)	0.0180 (6)	0.0170 (6)	−0.0035 (5)	−0.0014 (5)	−0.0014 (5)
C21	0.0155 (6)	0.0144 (6)	0.0149 (6)	−0.0043 (5)	−0.0007 (5)	0.0005 (5)
C22	0.0161 (6)	0.0143 (6)	0.0152 (6)	−0.0033 (5)	−0.0015 (5)	−0.0008 (5)
C23	0.0168 (6)	0.0146 (6)	0.0169 (6)	−0.0011 (5)	−0.0009 (5)	−0.0047 (5)
N23	0.0152 (6)	0.0203 (6)	0.0279 (7)	−0.0029 (5)	−0.0021 (5)	0.0022 (5)
C24	0.0142 (6)	0.0170 (6)	0.0176 (6)	−0.0036 (5)	0.0026 (5)	−0.0016 (5)
N25	0.0131 (5)	0.0148 (5)	0.0150 (5)	−0.0036 (4)	0.0008 (4)	0.0010 (4)
C25	0.0200 (7)	0.0154 (6)	0.0174 (6)	−0.0057 (5)	−0.0015 (5)	−0.0028 (5)
O25	0.0223 (5)	0.0202 (5)	0.0235 (5)	−0.0040 (4)	−0.0070 (4)	0.0036 (4)
O26	0.0213 (5)	0.0238 (5)	0.0187 (5)	−0.0064 (4)	−0.0001 (4)	0.0070 (4)
C26	0.0296 (8)	0.0247 (8)	0.0206 (7)	−0.0071 (6)	0.0005 (6)	0.0087 (6)
C27	0.0338 (9)	0.0273 (8)	0.0249 (8)	−0.0096 (7)	0.0058 (7)	0.0022 (6)
C28	0.0139 (6)	0.0197 (6)	0.0114 (6)	−0.0033 (5)	−0.0007 (5)	−0.0012 (5)
C29	0.0154 (6)	0.0189 (7)	0.0166 (6)	−0.0019 (5)	0.0011 (5)	0.0006 (5)
C30	0.0203 (7)	0.0214 (7)	0.0193 (7)	0.0002 (5)	−0.0014 (5)	0.0013 (5)
O30	0.0195 (6)	0.0309 (6)	0.0468 (7)	0.0014 (5)	0.0085 (5)	0.0048 (5)
O31	0.0251 (6)	0.0197 (5)	0.0367 (6)	0.0048 (4)	0.0035 (5)	0.0024 (5)
C31	0.0335 (9)	0.0267 (9)	0.0482 (11)	0.0124 (7)	0.0030 (8)	−0.0003 (8)
C32	0.0143 (6)	0.0180 (6)	0.0175 (6)	−0.0011 (5)	0.0005 (5)	0.0001 (5)
O32	0.0184 (5)	0.0302 (6)	0.0236 (5)	−0.0091 (4)	−0.0031 (4)	−0.0008 (4)
O33	0.0200 (5)	0.0275 (5)	0.0157 (5)	−0.0086 (4)	0.0040 (4)	−0.0010 (4)
C33	0.0280 (8)	0.0453 (10)	0.0247 (8)	−0.0138 (7)	0.0115 (7)	0.0005 (7)

### Geometric parameters (Å, º)

**Table:**

C1—N25	1.4751 (16)	C19—H19	0.9500
C1—C2	1.5210 (18)	C20—C21	1.5489 (18)
C1—C24	1.5383 (18)	C21—N25	1.4706 (17)
C1—H1	1.0000	C21—C22	1.5150 (18)
C2—C3	1.3916 (19)	C21—H21	1.0000
C2—C7	1.3970 (19)	C22—C23	1.3671 (18)
C3—C4	1.389 (2)	C22—C25	1.4437 (19)
C3—H3	0.9500	C23—N23	1.3458 (18)
C4—C5	1.386 (2)	C23—C24	1.4983 (19)
C4—H4	0.9500	N23—H23A	0.891 (18)
C5—C6	1.386 (2)	N23—H23B	0.936 (18)
C5—H5	0.9500	C24—H24A	0.9900
C6—C7	1.389 (2)	C24—H24B	0.9900
C6—H6	0.9500	N25—C28	1.3551 (17)
C7—O8	1.3891 (17)	C25—O25	1.2284 (17)
O8—C9	1.4354 (17)	C25—O26	1.3546 (17)
C9—C10	1.504 (2)	O26—C26	1.4566 (17)
C9—H9A	0.9900	C26—C27	1.501 (2)
C9—H9B	0.9900	C26—H26A	0.9900
C10—O11	1.4352 (17)	C26—H26B	0.9900
C10—H10A	0.9900	C27—H27A	0.9800
C10—H10B	0.9900	C27—H27B	0.9800
O11—C12	1.4195 (18)	C27—H27C	0.9800
C12—C13	1.505 (2)	C28—C29	1.3555 (19)
C12—H12A	0.9900	C28—C32	1.5172 (19)
C12—H12B	0.9900	C29—C30	1.4488 (19)
C13—O14	1.4348 (16)	C29—H29	0.9500
C13—H13A	0.9900	C30—O30	1.2083 (18)
C13—H13B	0.9900	C30—O31	1.3590 (18)
O14—C15	1.3765 (16)	O31—C31	1.4353 (18)
C15—C16	1.4008 (19)	C31—H31A	0.9800
C15—C20	1.4039 (19)	C31—H31B	0.9800
C16—C17	1.387 (2)	C31—H31C	0.9800
C16—H16	0.9500	C32—O32	1.2058 (16)
C17—C18	1.380 (2)	C32—O33	1.3292 (16)
C17—H17	0.9500	O33—C33	1.4448 (17)
C18—C19	1.387 (2)	C33—H33A	0.9800
C18—H18	0.9500	C33—H33B	0.9800
C19—C20	1.3950 (19)	C33—H33C	0.9800
			
N25—C1—C2	111.41 (10)	C15—C20—C21	127.59 (12)
N25—C1—C24	110.41 (10)	N25—C21—C22	109.45 (10)
C2—C1—C24	111.32 (11)	N25—C21—C20	115.95 (11)
N25—C1—H1	107.8	C22—C21—C20	111.71 (11)
C2—C1—H1	107.8	N25—C21—H21	106.4
C24—C1—H1	107.8	C22—C21—H21	106.4
C3—C2—C7	118.01 (12)	C20—C21—H21	106.4
C3—C2—C1	122.28 (12)	C23—C22—C25	120.74 (12)
C7—C2—C1	119.70 (12)	C23—C22—C21	117.09 (12)
C4—C3—C2	121.00 (14)	C25—C22—C21	121.85 (12)
C4—C3—H3	119.5	N23—C23—C22	125.65 (13)
C2—C3—H3	119.5	N23—C23—C24	117.38 (12)
C5—C4—C3	120.18 (14)	C22—C23—C24	116.96 (12)
C5—C4—H4	119.9	C23—N23—H23A	116.4 (11)
C3—C4—H4	119.9	C23—N23—H23B	119.3 (11)
C4—C5—C6	119.75 (14)	H23A—N23—H23B	122.3 (16)
C4—C5—H5	120.1	C23—C24—C1	112.42 (11)
C6—C5—H5	120.1	C23—C24—H24A	109.1
C5—C6—C7	119.77 (14)	C1—C24—H24A	109.1
C5—C6—H6	120.1	C23—C24—H24B	109.1
C7—C6—H6	120.1	C1—C24—H24B	109.1
C6—C7—O8	119.54 (13)	H24A—C24—H24B	107.9
C6—C7—C2	121.28 (13)	C28—N25—C21	121.49 (11)
O8—C7—C2	119.08 (12)	C28—N25—C1	118.50 (11)
C7—O8—C9	115.23 (11)	C21—N25—C1	119.36 (10)
O8—C9—C10	108.14 (12)	O25—C25—O26	121.82 (13)
O8—C9—H9A	110.1	O25—C25—C22	124.65 (13)
C10—C9—H9A	110.1	O26—C25—C22	113.49 (12)
O8—C9—H9B	110.1	C25—O26—C26	116.03 (11)
C10—C9—H9B	110.1	O26—C26—C27	106.66 (12)
H9A—C9—H9B	108.4	O26—C26—H26A	110.4
O11—C10—C9	109.54 (12)	C27—C26—H26A	110.4
O11—C10—H10A	109.8	O26—C26—H26B	110.4
C9—C10—H10A	109.8	C27—C26—H26B	110.4
O11—C10—H10B	109.8	H26A—C26—H26B	108.6
C9—C10—H10B	109.8	C26—C27—H27A	109.5
H10A—C10—H10B	108.2	C26—C27—H27B	109.5
C12—O11—C10	113.88 (11)	H27A—C27—H27B	109.5
O11—C12—C13	109.95 (12)	C26—C27—H27C	109.5
O11—C12—H12A	109.7	H27A—C27—H27C	109.5
C13—C12—H12A	109.7	H27B—C27—H27C	109.5
O11—C12—H12B	109.7	N25—C28—C29	125.26 (12)
C13—C12—H12B	109.7	N25—C28—C32	115.09 (11)
H12A—C12—H12B	108.2	C29—C28—C32	119.51 (12)
O14—C13—C12	109.07 (12)	C28—C29—C30	121.04 (13)
O14—C13—H13A	109.9	C28—C29—H29	119.5
C12—C13—H13A	109.9	C30—C29—H29	119.5
O14—C13—H13B	109.9	O30—C30—O31	122.38 (13)
C12—C13—H13B	109.9	O30—C30—C29	127.43 (14)
H13A—C13—H13B	108.3	O31—C30—C29	110.18 (12)
C15—O14—C13	116.89 (11)	C30—O31—C31	115.59 (13)
O14—C15—C16	121.72 (12)	O31—C31—H31A	109.5
O14—C15—C20	118.30 (12)	O31—C31—H31B	109.5
C16—C15—C20	119.97 (13)	H31A—C31—H31B	109.5
C17—C16—C15	120.61 (13)	O31—C31—H31C	109.5
C17—C16—H16	119.7	H31A—C31—H31C	109.5
C15—C16—H16	119.7	H31B—C31—H31C	109.5
C18—C17—C16	120.25 (13)	O32—C32—O33	125.66 (13)
C18—C17—H17	119.9	O32—C32—C28	125.19 (12)
C16—C17—H17	119.9	O33—C32—C28	109.06 (11)
C17—C18—C19	118.88 (14)	C32—O33—C33	115.97 (11)
C17—C18—H18	120.6	O33—C33—H33A	109.5
C19—C18—H18	120.6	O33—C33—H33B	109.5
C18—C19—C20	122.76 (14)	H33A—C33—H33B	109.5
C18—C19—H19	118.6	O33—C33—H33C	109.5
C20—C19—H19	118.6	H33A—C33—H33C	109.5
C19—C20—C15	117.52 (12)	H33B—C33—H33C	109.5
C19—C20—C21	114.89 (12)		
			
N25—C1—C2—C3	54.79 (17)	N25—C21—C22—C25	127.01 (13)
C24—C1—C2—C3	−68.92 (16)	C20—C21—C22—C25	−103.19 (14)
N25—C1—C2—C7	−126.67 (13)	C25—C22—C23—N23	7.7 (2)
C24—C1—C2—C7	109.61 (14)	C21—C22—C23—N23	−178.62 (12)
C7—C2—C3—C4	−0.7 (2)	C25—C22—C23—C24	−170.83 (12)
C1—C2—C3—C4	177.82 (13)	C21—C22—C23—C24	2.85 (17)
C2—C3—C4—C5	1.3 (2)	N23—C23—C24—C1	−134.29 (12)
C3—C4—C5—C6	−0.6 (2)	C22—C23—C24—C1	44.37 (16)
C4—C5—C6—C7	−0.6 (2)	N25—C1—C24—C23	−44.58 (14)
C5—C6—C7—O8	177.57 (13)	C2—C1—C24—C23	79.70 (14)
C5—C6—C7—C2	1.2 (2)	C22—C21—N25—C28	−145.08 (12)
C3—C2—C7—C6	−0.5 (2)	C20—C21—N25—C28	87.47 (14)
C1—C2—C7—C6	−179.10 (12)	C22—C21—N25—C1	44.29 (15)
C3—C2—C7—O8	−176.89 (12)	C20—C21—N25—C1	−83.17 (14)
C1—C2—C7—O8	4.51 (19)	C2—C1—N25—C28	64.94 (15)
C6—C7—O8—C9	87.42 (16)	C24—C1—N25—C28	−170.83 (11)
C2—C7—O8—C9	−96.13 (15)	C2—C1—N25—C21	−124.15 (12)
C7—O8—C9—C10	151.09 (12)	C24—C1—N25—C21	0.08 (15)
O8—C9—C10—O11	−70.39 (15)	C23—C22—C25—O25	−13.2 (2)
C9—C10—O11—C12	127.74 (13)	C21—C22—C25—O25	173.47 (12)
C10—O11—C12—C13	179.42 (11)	C23—C22—C25—O26	164.83 (12)
O11—C12—C13—O14	73.13 (15)	C21—C22—C25—O26	−8.55 (18)
C12—C13—O14—C15	−172.13 (12)	O25—C25—O26—C26	3.11 (19)
C13—O14—C15—C16	−13.40 (18)	C22—C25—O26—C26	−174.93 (12)
C13—O14—C15—C20	167.47 (12)	C25—O26—C26—C27	172.59 (12)
O14—C15—C16—C17	−179.30 (13)	C21—N25—C28—C29	−165.86 (12)
C20—C15—C16—C17	−0.2 (2)	C1—N25—C28—C29	4.85 (19)
C15—C16—C17—C18	0.5 (2)	C21—N25—C28—C32	18.46 (17)
C16—C17—C18—C19	−0.5 (2)	C1—N25—C28—C32	−170.83 (11)
C17—C18—C19—C20	0.1 (2)	N25—C28—C29—C30	−170.74 (13)
C18—C19—C20—C15	0.2 (2)	C32—C28—C29—C30	4.76 (19)
C18—C19—C20—C21	−179.26 (13)	C28—C29—C30—O30	−6.7 (2)
O14—C15—C20—C19	178.97 (12)	C28—C29—C30—O31	172.40 (13)
C16—C15—C20—C19	−0.2 (2)	O30—C30—O31—C31	0.2 (2)
O14—C15—C20—C21	−1.6 (2)	C29—C30—O31—C31	−178.94 (13)
C16—C15—C20—C21	179.24 (13)	N25—C28—C32—O32	−90.53 (17)
C19—C20—C21—N25	−170.28 (11)	C29—C28—C32—O32	93.52 (18)
C15—C20—C21—N25	10.30 (19)	N25—C28—C32—O33	86.20 (14)
C19—C20—C21—C22	63.39 (15)	C29—C28—C32—O33	−89.74 (15)
C15—C20—C21—C22	−116.04 (15)	O32—C32—O33—C33	−8.0 (2)
N25—C21—C22—C23	−46.59 (15)	C28—C32—O33—C33	175.27 (12)
C20—C21—C22—C23	83.21 (14)		

### Hydrogen-bond geometry (Å, º)

**Table:**

*D*—H···*A*	*D*—H	H···*A*	*D*···*A*	*D*—H···*A*
C1—H1···O11	1.00	2.38	3.2572 (17)	146
N23—H23*A*···O25	0.891 (18)	2.040 (18)	2.7127 (17)	131.4 (15)
N23—H23*B*···O32^i^	0.936 (18)	2.063 (18)	2.9986 (17)	176.7 (16)
C29—H29···O8	0.95	2.44	3.3439 (17)	159

Symmetry code: (i) *x*+1, *y*, *z*.

## References

[bb1] Anh, L. T., Hieu, T. H., Soldatenkov, A. T., Kolyadina, N. M. & Khrustalev, V. N. (2012*b*). *Acta Cryst.* E**68**, o1588–o1589. 10.1107/S1600536812018867 PMC3379201 22719399

[bb2] Anh, L. T., Hieu, T. H., Soldatenkov, A. T., Kolyadina, N. M. & Khrustalev, V. N. (2012*c*). *Acta Cryst.* E**68**, o2165–o2166. 10.1107/S1600536812027274 PMC3393972 22798837

[bb3] Anh, L. T., Hieu, T. H., Soldatenkov, A. T., Soldatova, S. A. & Khrustalev, V. N. (2012*a*). *Acta Cryst.* E**68**, o1386–o1387. 10.1107/S1600536812015206 PMC3344514 22590276

[bb4] Anh, L. T., Levov, A. N., Soldatenkov, A. T., Gruzdev, R. D. & Hieu, T. H. (2008). *Russ. J. Org. Chem.* **44**, 463–465.

[bb5] Anh, L. T., Phuong, N. T. T., Hieu, T. H., Soldatenkov, A. T., Van, B. T., Van, T. T. T., Nhung, D. T., Voskressensky, L. G., Tung, T. H. & Khrustalev, V. N. (2018). *Macroheterocycles*, **11**, 197–202.

[bb6] Bradshaw, J. S. & Izatt, R. M. (1997). *Acc. Chem. Res.* **30**, 338–345.

[bb7] Bruker (2001). *SAINT*. Bruker AXS Inc., Madison, Wisconsin, USA.

[bb8] Bruker (2005). *APEX2*. Bruker AXS Inc., Madison, Wisconsin, USA.

[bb9] Gokel, G. W. & Murillo, O. (1996). *Acc. Chem. Res.* **29**, 425–432.

[bb10] Hieu, T. H., Anh, L. T., Soldatenkov, A. T., Golovtsov, N. I. & Soldatova, S. A. (2011). *Chem. Heterocyc. Compd*, **47**, 1307–1308.

[bb11] Hieu, T. H., Anh, L. T., Soldatenkov, A. T., Gorchakova, O. S. & Khrustalev, V. N. (2013*b*). *Acta Cryst.* E**69**, o1023–o1024. 10.1107/S1600536813014748 PMC3772461 24046604

[bb12] Hieu, T. H., Anh, L. T., Soldatenkov, A. T., Kolyadina, N. M. & Khrustalev, V. N. (2012*a*). *Acta Cryst.* E**68**, o2431–o2432. 10.1107/S1600536812030644 PMC3414347 22904880

[bb13] Hieu, T. H., Anh, L. T., Soldatenkov, A. T., Kurilkin, V. V. & Khrustalev, V. N. (2012*b*). *Acta Cryst.* E**68**, o2848–o2849. 10.1107/S1600536812037051 PMC3470208 23125652

[bb14] Hieu, T. H., Anh, L. T., Soldatenkov, A. T., Vasil’ev, V. G. & Khrustalev, V. N. (2013*a*). *Acta Cryst.* E**69**, o565–o566. 10.1107/S1600536813007241 PMC3629615 23634102

[bb15] Hiraoka, M. (1978). *.* In *Crown Compounds: Their Characteristics and Application*. Tokyo: Kodansha.

[bb16] Khieu, T. H., Soldatenkov, A. T., Le Tuan, A., Levov, A. N., Smol’yakov, A. F., Khrustalev, V. N. & Antipin, M. Yu. (2011). *Russ. J. Org. Chem.* **47**, 766–770.

[bb17] Komarova, A. I., Levov, A. N., Soldatenkov, A. T. & Soldatova, S. A. (2008). *Chem. Heterocycl. C.* **44**, 624–625.

[bb18] Le, A. T., Truong, H. H., Nguyen, P. T. T., Pham, H. T., Kotsuba, V. E., Soldatenkov, A. T., Khrustalev, V. N. & Wodajo, A. T. (2014). *Macroheterocycles*, **7**, 386–390.

[bb19] Le, T. A., Truong, H. H., Thi, T. P. N., Thi, N. D., To, H. T., Thi, H. P. & Soldatenkov, A. T. (2015). *Mendeleev Commun.* **25**, 224–225.

[bb20] Levov, A. N., Anh, L. T., Komarova, A. I., Strokina, V. M., Soldatenkov, A. T. & Khrustalev, V. N. (2008*a*). *Russ. J. Org. Chem.* **44**, 457–462.

[bb21] Levov, A. N., Komarov, A. I., Soldatenkov, A. T., Avramenko, G. V., Soldatova, S. A. & Khrustalev, V. N. (2008*b*). *Russ. J. Org. Chem.* **44**, 1665–1670.

[bb22] Levov, A. N., Strokina, V. M., Anh, L. T., Komarova, A. I., Soldatenkov, A. T. & Khrustalev, V. N. (2006). *Mendeleev Commun.* **16**, 35–36.

[bb23] Pedersen, C. J. (1988). *Angew. Chem.* **100**, 1053–1059.

[bb24] Sadym, A., Lagunin, A., Filimonov, D. & Poroikov, V. (2003). *SAR QSAR Environ. Res.* **14**, 339–347. 10.1080/10629360310001623935 14758978

[bb28] Schwan, A. L. & Warkentin, J. (1988). *Can. J. Chem.* **66**, 1686–1694.

[bb25] Sheldrick, G. M. (2003). *SADABS*. University of Göttingen, Germany

[bb26] Sheldrick, G. M. (2015). *Acta Cryst.* C**71**, 3–8.

[bb27] Sokol, V. I., Kolyadina, N. M., Kvartalov, V. B., Sergienko, V. S., Soldatenkov, A. T. & Davydov, V. V. (2011). *Russ. Chem. Bull.* **60**, 2086–2088.

